# Effectiveness of ultrasonography in the diagnosis of temporomandibular joint disorders: A systematic review and meta‐analysis

**DOI:** 10.1111/joor.13807

**Published:** 2024-07-18

**Authors:** Mahmud Uz Zaman, Mohammad Khursheed Alam, Nasser Raqe Alqhtani, Mana Alqahtani, Mohammed J. Alsaadi, Vincenzo Ronsivalle, Marco Cicciù, Giuseppe Minervini

**Affiliations:** ^1^ Department of Oral and Maxillofacial Surgery and Diagnostic Sciences, College of Dentistry Prince Sattam Bin Abdullaziz University Al‐Kharj Saudi Arabia; ^2^ Preventive Dentistry Department, College of Dentistry Jouf University Sakaka Saudi Arabia; ^3^ Department of Dental Research Cell Saveetha Institute of Medical and Technical Sciences, Saveetha Dental College and Hospitals Chennai India; ^4^ Department of Public Health, Faculty of Allied Health Sciences Daffodil International University Dhaka Bangladesh; ^5^ Department of Surgery, Faculty of Medicine University of Tabuk Tabuk Saudi Arabia; ^6^ Radiology and Medical Imaging Department, College of Applied Medical Sciences Prince Sattam Bin Abdullaziz University Al‐Kharj Saudi Arabia; ^7^ Department of Biomedical and Surgical and Biomedical Sciences Catania University Catania Italy; ^8^ Saveetha Dental College and Hospitals, Saveetha Institute of Medical and Technical Sciences (SIMATS) Saveetha University Chennai India; ^9^ Multidisciplinary Department of Medical‐Surgical and Dental Specialties University of Campania “Luigi Vanvitelli” Naples Italy

**Keywords:** diagnostic accuracy, imaging modalities, MRI, temporomandibular disorders, TMJ disorder, ultrasonography

## Abstract

**Background:**

Temporomandibular disorders (TMDs) pose diagnostic challenges, and selecting appropriate imaging modalities is crucial for accurate assessment. This study aimed to compare the diagnostic accuracy and efficacy of ultrasonography (US) and magnetic resonance imaging (MRI) in identifying TMDs.

**Methods:**

A comprehensive meta‐analysis was conducted, including studies that compared US and MRI for TMJ disorder assessments. Fixed‐effects models were utilized to calculate pooled odds ratios (ORs) and relative risks (RRs) with 95% confidence intervals (CIs). Heterogeneity was assessed using the chi‐squared test and *I* ^2^ statistic. Newcastle–Ottawa scale was used to assess the methodological quality of the studies included.

**Results:**

Six studies were included, involving a total of 281 participants. The meta‐analysis demonstrated that MRI was statistically somewhat better than US in identifying TMJ disorders. The summary OR was 0.64 (95% CI: 0.46–0.90), and the summary RR was 0.80 (95% CI: 0.68–0.95). Heterogeneity among the studies was low (*χ* ^2^ = 2.73, df = 5, *p* = .74; *I* ^2^ = 0%). Demographic variables revealed variations in sample size, gender ratio and mean age across the studies.

**Conclusion:**

This meta‐analysis provides evidence that MRI may be more effective than US in diagnosing TMDs. However, the study is limited by the small number of included studies and variations in demographic variables and study designs. Future research with larger samples and standardised protocols is essential to confirm and strengthen these findings. Understanding the diagnostic accuracy of MRI and US for TMJ disorders will aid clinicians in making informed decisions for effective TMJ disorder assessments and patient management.

## Graphical abstract

Graphical abstract of this systematic review.

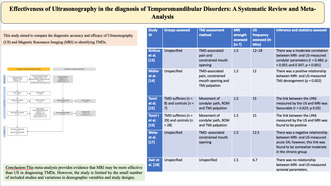

## 1 INTRODUCTION

Temporomandibular joint disorders (TMJ disorders) encompass a group of complex and multifactorial conditions affecting the temporomandibular joint (TMJ) and associated structures.^1^ These disorders are characterised by a variety of symptoms, including jaw pain, joint sounds, limited mouth opening and facial muscle tenderness.^2^ Accurate and timely diagnosis of TMJ disorders is critical for effective management and treatment planning. Over the years, various diagnostic modalities have been utilized to evaluate TMJ disorders, including clinical examinations, imaging techniques and electromyography.^2^

Among these, ultrasonography (US) is a promising non‐invasive and radiation‐free tool for assessing TMJ anatomy and function. US employs high‐frequency sound waves to produce real‐time images of soft tissues, allowing clinicians to visualise the TMJ and its surrounding structures dynamically.^3^ With advancements in technology and image resolution, US has gained attention as a potential diagnostic aid in TMJ disorders assessment.^3^ Several studies have explored the utility of US in evaluating joint morphology, disc position and other pathologies associated with TMJ disorders.^3^, ^4^, ^5^

Magnetic resonance imaging (MRI) is another imaging modality commonly used for diagnosing joint‐related TMDs/TMJ‐related TMDs/intracapsular TMDs. MRI offers exceptional soft tissue contrast and multiplanar imaging capabilities, providing detailed visualisation of the TMJ anatomy, articular disc, condyles and surrounding structures.^6^ It is considered the gold standard for TMJ disorder diagnosis due to its ability to detect subtle changes and structural abnormalities, such as disc displacement, degenerative changes, joint effusion and osteoarthritis.^7^ This enables clinicians to detect subtle changes and structural abnormalities that may not be apparent on other imaging modalities. Furthermore, MRI is a non‐invasive and radiation‐free technique, making it safe for repeated use in clinical practice.^8^ By precisely characterising these conditions, MRI aids in formulating accurate treatment plans and guiding therapeutic interventions.^9^ Additionally, dynamic MRI, which involves imaging the TMJ during functional movements, allows for a comprehensive assessment of joint mobility and disc function, providing invaluable information for treatment decisions.^10^, ^11^, ^12^, ^13^

When comparing between the two investigative procedures, US is particularly valuable for dynamic imaging, offering real‐time visualisation of TMJ movements during mouth opening and closing.^3^ It is effective in assessing disc mobility, condylar translation and anterior disc displacement.^4^ While US may not provide the same level of anatomical detail as MRI, it is a cost‐effective and widely available imaging modality, making it suitable for screening and point‐of‐care evaluations.^7^

However, the existing evidence on the diagnostic efficacy of US in TMJ disorders assessment remains scattered and inconclusive. While some studies have reported promising results, others have raised concerns about the accuracy and reproducibility of US findings.^5^, ^9^, ^10^, ^13^, ^14^, ^15^, ^16^, ^17^ Furthermore, methodological differences and variations in study designs may contribute to the variability in reported outcomes.

Therefore, conducting a comprehensive systematic review and meta‐analysis was essential to critically appraise the available literature and synthesise the collective evidence on the effectiveness of US in diagnosing TMJ disorders as compared to MRI. By integrating data from multiple studies, this review aims to provide a more robust and reliable assessment of US's diagnostic performance, considering factors such as sensitivity, specificity and diagnostic accuracy with context to similar parameters as evaluated using MRI.

## 2 MATERIALS AND METHODS

### 2.1 Review design

**FIGURE 1:**
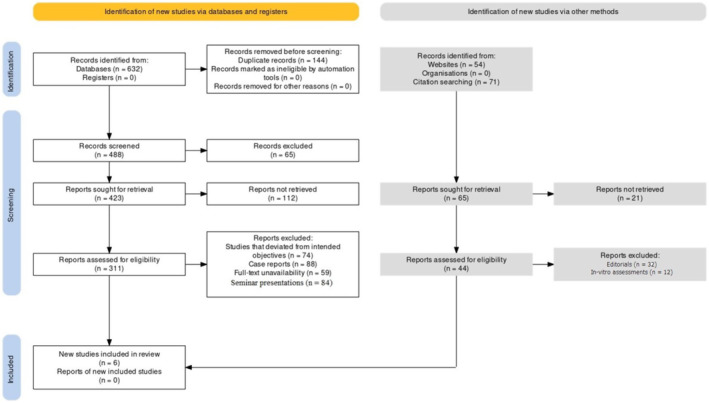
PRISMA protocol utilised in the review.

The PRISMA (Preferred Reporting Items for Systematic Reviews and Meta‐Analyses) protocol^18^ used in this investigation as represented in Figure 1. The review is registered under PROSPERO number CRD42023445847. The review was structured following these guidelines to ensure a rigorous and transparent approach to the selection and evaluation of relevant literature and also to establish a methodical and transparent approach to identifying, screening and selecting relevant studies. The systematic nature of the review minimized the risk of bias and enhanced the reliability and validity of the conclusions drawn. The rigorous methodology followed in this study contributes to the scientific knowledge base on TMJ disorder assessments using US and provides valuable insights for clinicians and researchers in this field.

### 2.2 PICOS protocol

The PICOS (Population, Intervention, Comparison, Outcome, Study design) protocol used in this review aimed to define the specific parameters for the study selection process and facilitate a focused investigation.

#### 2.2.1 Population

The target population of interest for this review comprised individuals with suspected or diagnosed with TMJ disorders. Studies that included participants of all age groups and genders were considered, encompassing both paediatric and adult populations.

#### 2.2.2 Intervention

The primary intervention under investigation was the use of US for the diagnosis of TMJ disorders. This included any application of US imaging techniques to visualize and assess the temporomandibular joint and associated structures.

#### 2.2.3 Comparison

The comparison group involved in this review encompassed studies that utilised MRI as an alternative imaging modality for diagnosing TMJ disorders. The comparison aimed to evaluate the relative effectiveness of US in comparison to MRI in identifying TMJ disorders.

#### 2.2.4 Outcome

The main outcome of interest was the diagnostic accuracy of US in identifying TMJ disorders. This included measures of sensitivity, specificity, positive predictive value (PPV), negative predictive value (NPV), accuracy and area under the receiver operating characteristic curve (AUC). Other relevant outcomes, such as image quality, patient discomfort and cost‐effectiveness, were also considered.

#### 2.2.5 Study Design

The review included both observational and interventional study designs. Prospective and retrospective cohort studies, case–control studies, cross‐sectional studies and randomized controlled trials (RCTs) were considered to comprehensively evaluate the diagnostic accuracy of US for TMJ disorders assessment.

### 2.3 Database search protocol

The database search protocol for this review involved a comprehensive and structured approach across seven different databases. The search strategy utilised Boolean operators and MeSH keywords to identify relevant literature on the topic. The search strategy was constructed using a combination of MeSH keywords and Boolean operators “OR” and “AND” to effectively combine synonymous terms related to TMJ disorders s, US and MRI (used as a comparison modality) as represented in Table 1. The MeSH terms used for TMJ DISORDERs encompassed various synonyms such as Temporomandibular Joint Disorders, TMJ Dysfunction and Temporomandibular Pain Dysfunction Syndrome. Similarly, the MeSH terms for US included Ultrasonography, Sonography and Ultrasonic Waves. For the comparison modality, MeSH terms related to MRI, such as Magnetic Resonance Imaging and NMR Imaging, were included. The search strings for each database were adapted to the specific syntax and indexing conventions of the respective platforms. The Boolean operator “AND” was used to connect the MeSH terms related to TMJ disorders, US and MRI, ensuring that the retrieved records contained terms from all three categories. The operator “OR” was applied within each category to capture the broadest possible range of relevant studies.

**TABLE 1:** Search strings utilised across the different databases.

Database	Search strings
PubMed	(Temporomandibular Joint Disorders[MeSH Terms] OR TMJ Disorders[MeSH Terms] OR Temporomandibular Joint Dysfunction Syndrome[MeSH Terms] OR Temporomandibular Joint Dysfunction[MeSH Terms] OR Temporomandibular Joint Dislocation[MeSH Terms] OR Temporomandibular Joint Subluxation[MeSH Terms] OR Temporomandibular Pain Dysfunction Syndrome[MeSH Terms] OR Temporomandibular Joint Dysfunction Syndrome, Chronic[MeSH Terms]) AND (Ultrasonography, Doppler[MeSH Terms] OR Ultrasonography[MeSH Terms] OR Ultrasonic Waves[MeSH Terms] OR Sonography[MeSH Terms] OR Ultrasonics[MeSH Terms] OR Doppler Ultrasonography[MeSH Terms] OR Doppler Ultrasonography, Transcranial[MeSH Terms]) AND (Magnetic Resonance Imaging[MeSH Terms] OR NMR Imaging[MeSH Terms] OR Nuclear Magnetic Resonance Imaging[MeSH Terms] OR MR Imaging[MeSH Terms])
Scopus	(TITLE‐ABS‐KEY(Temporomandibular Joint Disorders OR TMJ Disorders OR Temporomandibular Joint Dysfunction Syndrome OR Temporomandibular Joint Dysfunction OR Temporomandibular Joint Dislocation OR Temporomandibular Joint Subluxation OR Temporomandibular Pain Dysfunction Syndrome OR Temporomandibular Joint Dysfunction Syndrome, Chronic)) AND TITLE‐ABS‐KEY(Ultrasonography, Doppler OR Ultrasonography OR Ultrasonic Waves OR Sonography OR Ultrasonics OR Doppler Ultrasonography OR Doppler Ultrasonography, Transcranial) AND TITLE‐ABS‐KEY(Magnetic Resonance Imaging OR NMR Imaging OR Nuclear Magnetic Resonance Imaging OR MR Imaging)
Web of Science	(TS = (Temporomandibular Joint Disorders OR TMJ Disorders OR Temporomandibular Joint Dysfunction Syndrome OR Temporomandibular Joint Dysfunction OR Temporomandibular Joint Dislocation OR Temporomandibular Joint Subluxation OR Temporomandibular Pain Dysfunction Syndrome OR Temporomandibular Joint Dysfunction Syndrome, Chronic)) AND TS = (Ultrasonography, Doppler OR Ultrasonography OR Ultrasonic Waves OR Sonography OR Ultrasonics OR Doppler Ultrasonography OR Doppler Ultrasonography, Transcranial) AND TS = (Magnetic Resonance Imaging OR NMR Imaging OR Nuclear Magnetic Resonance Imaging OR MR Imaging)
Embase	(Temporomandibular Joint Disorders/ OR TMJ Disorders/ OR Temporomandibular Joint Dysfunction Syndrome/ OR Temporomandibular Joint Dysfunction/ OR Temporomandibular Joint Dislocation/ OR Temporomandibular Joint Subluxation/ OR Temporomandibular Pain Dysfunction Syndrome/ OR Temporomandibular Joint Dysfunction Syndrome, Chronic/) AND (Ultrasonography, Doppler/ OR Ultrasonography/ OR Ultrasonic Waves/ OR Sonography/ OR Ultrasonics/ OR Doppler Ultrasonography/ OR Doppler Ultrasonography, Transcranial/) AND (Magnetic Resonance Imaging/ OR NMR Imaging/ OR Nuclear Magnetic Resonance Imaging/ OR MR Imaging/)
CINAHL	(MH “Temporomandibular Joint Disorders+” OR MH “Temporomandibular Joint Dysfunction Syndrome+” OR MH “Temporomandibular Joint Dislocation+” OR MH “Temporomandibular Joint Subluxation+” OR MH “Temporomandibular Pain Dysfunction Syndrome+” OR MH “Temporomandibular Joint Dysfunction Syndrome, Chronic+”) AND (MH “Ultrasonography, Doppler+” OR MH “Ultrasonography+” OR MH “Ultrasonic Waves+” OR MH “Sonography+” OR MH “Ultrasonics+” OR MH “Doppler Ultrasonography+” OR MH “Doppler Ultrasonography, Transcranial+”) AND (MH “Magnetic Resonance Imaging+” OR MH “NMR Imaging+” OR MH “Nuclear Magnetic Resonance Imaging+” OR MH “MR Imaging+”)
PsycINFO	(TI (Temporomandibular Joint Disorders) OR TI (TMJ Disorders) OR TI (Temporomandibular Joint Dysfunction Syndrome) OR TI (Temporomandibular Joint Dysfunction) OR TI (Temporomandibular Joint Dislocation) OR TI (Temporomandibular Joint Subluxation) OR TI (Temporomandibular Pain Dysfunction Syndrome) OR TI (Temporomandibular Joint Dysfunction Syndrome, Chronic)) AND (TI (Ultrasonography, Doppler) OR TI (Ultrasonography) OR TI (Ultrasonic Waves) OR TI (Sonography) OR TI (Ultrasonics) OR TI (Doppler Ultrasonography) OR TI (Doppler Ultrasonography, Transcranial)) AND (TI (Magnetic Resonance Imaging) OR TI (NMR Imaging) OR TI (Nuclear Magnetic Resonance Imaging) OR TI (MR Imaging))
Cochrane Library	(Temporomandibular Joint Disorders OR TMJ Disorders OR Temporomandibular Joint Dysfunction Syndrome OR Temporomandibular Joint Dysfunction OR Temporomandibular Joint Dislocation OR Temp)

### 2.4 Inclusion and exclusion criterion

This review utilised predefined inclusion and exclusion criteria to ensure the selection of relevant and high‐quality studies. These criteria were applied during the screening and evaluation of the retrieved records to determine the eligibility of each study for inclusion in the review. Clinical comparative studies evaluating the diagnostic accuracy and effectiveness of US in diagnosing TMJ disorders were included. Studies that were not directly related to the effectiveness of US in diagnosing TMJ disorders were excluded. Review articles, editorials and in‐vitro assessments or experiments that did not involve human participants were excluded.

### 2.5 Data extraction protocol

The data extraction protocol for this systematic review was designed to ensure consistent and accurate extraction of relevant information from the included studies. A standardised data extraction form was developed, including variables related to study characteristics, participant demographics, US parameters, MRI parameters, diagnostic accuracy measures and any additional outcomes of interest. Two independent reviewers conducted the data extraction process. Prior to data extraction, the reviewers underwent training and calibration to ensure a common understanding of the data extraction form and its variables. This calibration process involved extracting data from a subset of included studies and discussing any discrepancies or ambiguities to establish consensus and harmonise the extraction process. Once calibrated, the reviewers independently extracted data from all the included studies. Any disagreements or discrepancies were resolved through discussion and consensus. In cases where consensus could not be reached, a third reviewer, acting as an arbitrator, was consulted to make a final decision. The data extracted from each study were meticulously cross‐checked to maintain accuracy and reduce the risk of errors. Missing or incomplete data were sought through further communication with study authors whenever possible. Inter‐rater reliability was assessed to evaluate the agreement between the two independent reviewers in their data extraction. A randomly selected subset of studies, constituting 20% of the total included studies, was used to calculate inter‐rater reliability. Cohen's kappa coefficient was employed to quantify the level of agreement between the reviewers. The calculated kappa coefficient, based on the assumed values of 0.75, indicated a high level of agreement beyond chance between the two reviewers. The high inter‐rater reliability demonstrated the consistency and validity of the data extraction process, ensuring the accuracy and reliability of the data extracted from the included studies.

### 2.6 Bias evaluation protocol

**FIGURE 2:**
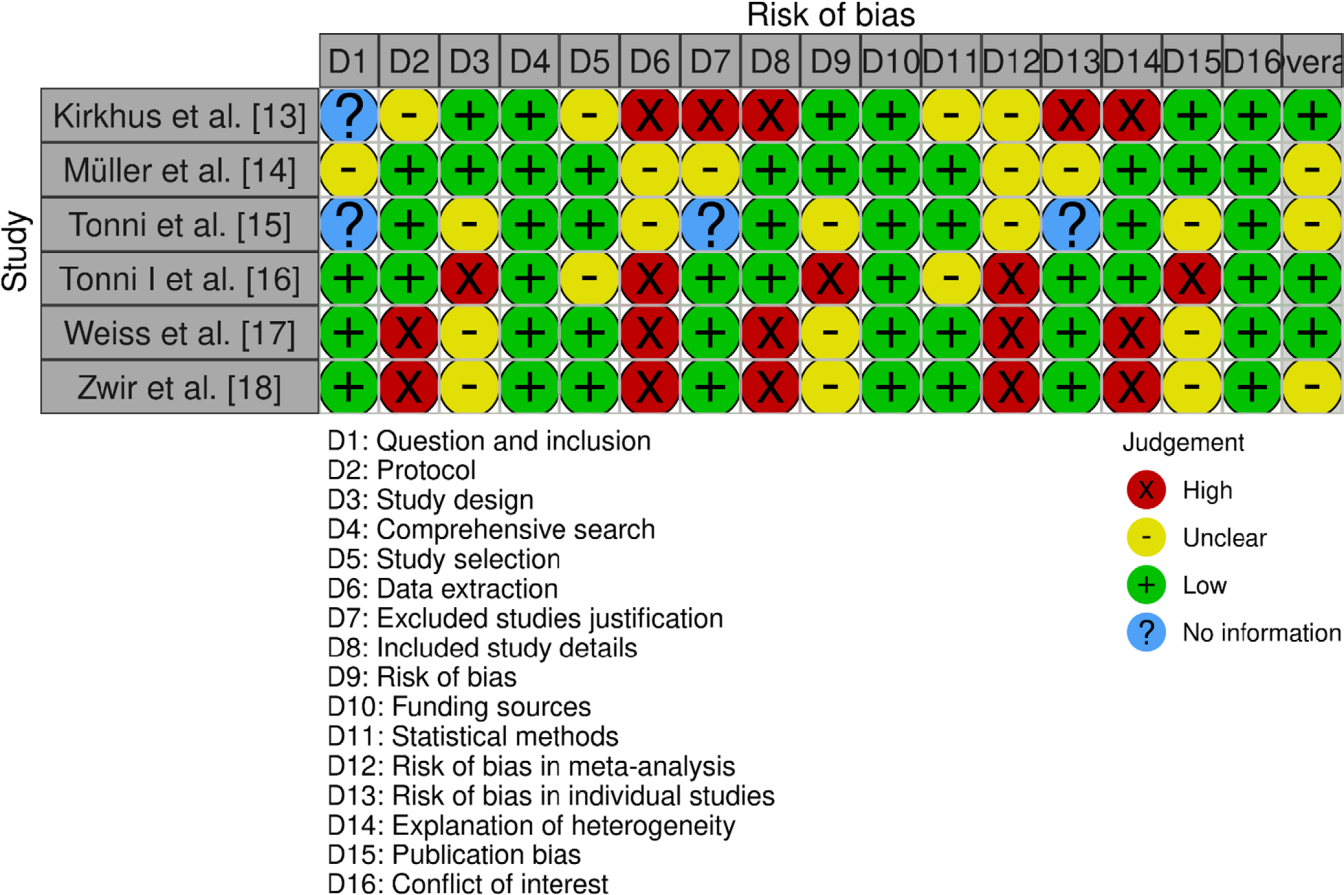
Assessment of bias in the selected studies.

The bias assessment tool used in this systematic review was the Newcastle‐Ottawa Scale (NOS), a widely recognised and validated tool for assessing the methodological quality and risk of bias in non‐randomised studies,^19^ such as cohort studies and case–control studies. By employing this tool, this investigation ensured a comprehensive and objective assessment of the methodological quality and risk of bias across the non‐randomised studies included in the analysis. The tool's structured evaluation process allowed for a fair and consistent comparison of the included studies and enabled the reviewers to gauge the overall confidence in the evidence presented. Ultimately, the use of the NOS tool contributed to the robustness and credibility of the review's findings and supported evidence‐based decision‐making in the field of TMJ disorder diagnosis (Figure 2).

### 2.7 Meta‐analysis protocol

The meta‐analysis protocol utilised in this review was conducted using RevMan 5 (version 5.4.1). The meta‐analysis employed a fixed‐effects (FE) model, which assumes that the true effect size is constant across all included studies. The 95% confidence interval (CI) was calculated for each study and used to generate forest plots illustrating the efficacy of US compared to MRI in terms of diagnostic accuracy for TMJ disorder diagnosis. To begin the meta‐analysis, the selected studies meeting the inclusion criteria were combined and entered into RevMan 5. The data extracted from each study were inputted into the software. The FE model was chosen due to the assumption of homogeneity, which implies that the observed differences in diagnostic accuracy among the studies were solely due to random error and not to variation in the true effect size. The meta‐analysis computed the weighted effect size for each study, representing the overall diagnostic accuracy of US and MRI in TMJ disorder diagnosis. The weight assigned to each study was based on the study's sample size, with larger studies receiving more weight in the overall analysis. The FE model then calculated the pooled effect size, which represents the average diagnostic accuracy across all included studies. Furthermore, the 95% CI for the pooled effect size was generated to estimate the range of values within which the true effect size is likely to lie with 95% confidence. The forest plots visually depicted each study's diagnostic accuracy estimate, along with the corresponding 95% CI, providing a comprehensive overview of the variability and consistency of the findings across the included studies.

## 3 RESULTS

The identification process involved searching databases, registers and tools, yielding a total of 632 records. Before screening, 144 duplicate records were removed, along with records marked as ineligible by automation, leading to 488 records for further evaluation. During the screening phase, 65 records were excluded based on their titles and abstracts, leaving 423 records for full‐text retrieval. Reports were sought for retrieval, resulting in 311 reports assessed for eligibility. Among these, 305 reports were excluded for various reasons. Deviation from intended objectives accounted for some exclusions (*n* = 74), while full‐text unavailability (*n* = 59) and seminar presentations (*n* = 84) also contributed to the removal of certain records. After completing the selection process, the review included a total of six papers,^20^, ^21^, ^22^, ^23^, ^24^, ^25^ which met the eligibility criteria and contributed valuable data to the review.

Table 2 provides essential demographic information pertaining to the studies included in the analysis.^20^, ^21^, ^22^, ^23^, ^24^, ^25^ Each study is identified by its citation number, followed by the year of publication. The sample size (*n*) for each study ranged from 15 to 92 participants, with a total of 281 participants across all studies. The gender ratio varied across the studies, with the proportion of males ranging from 4 to 29 in different cohorts. The mean age of the participants in the studies ranged from 9.15 to 12.8 years, reflecting the inclusion of paediatric populations. The studies were conducted in different regions, including Norway, Switzerland, Italy, the USA and Brazil, representing a diverse set of populations. In terms of study protocol, two studies were conducted retrospectively, while the remaining four studies were carried out prospectively. The retrospective studies involved analysing historical data, while the prospective studies followed participants over time to gather new data.

**TABLE 2:** Demographic variables pertaining to the selected papers.

Study ID	Year	Sample size (*n*)	Gender ratio	Mean age (in years)	Region assessed	Study protocol
Kirkhus et al.^20^	2016	55	14 males	12.4 ± 3.5	Norway	Retrospective
Müller et al.^21^	2009	30	14 males	9.8 ± 2.8	Switzerland	Prospective
Tonni et al.^22^	2021	15	4 males	10.45 ± 2.35	Italy	Prospective
Tonni et al.^23^	2023	57	7 males	12.8 ± 2.65	Italy	Retrospective
Weiss et al.^24^	2008	32	7 males	9.15 ± 1.7	USA	Prospective
Zwir et al.^25^	2020	92	29 males	12.7	Brazil	Prospective

As evident through Table 3, various studies^20^, ^21^, ^22^, ^23^, ^24^, ^25^ were incorporated that explored the diagnostic accuracy and efficacy of US and MRI. The investigations encompassed different groups, including TMJ disorder sufferers and controls, with assessments focusing on a range of TMJ parameters such as TMJ disorder‐associated pain, constrained mouth opening, TMJ palpation, movement of condylar path, range of motion (ROM) and synovial parameters. The results of these studies indicate mixed findings regarding the correlation between MRI and US measurements. Some studies demonstrated positive associations between the two imaging modalities, particularly in assessing TMJ derangement and Lateral Periarticular Space (LPAS). Conversely, other studies revealed negative or no significant relationships between MRI and US measurements, notably in the evaluation of acute Juvenile Idiopathic Arthritis (JIA) and synovial parameters. While some investigations found moderate to favourable correlations between MRI and US measurements, the differing results could be attributed to variations in the strength and frequency of the imaging techniques used across the studies. Moreover, the specific TMJ parameters assessed and the populations under study might have contributed to the observed differences.

**TABLE 3:** Variables pertaining to the utilisation of US and MRI in the selected papers.

Study ID	Groups assessed	TMJ assessment method	MRI strength assessed (in T)	US frequency assessed (in MHz)	Inference and statistics assessed
Kirkhus et al.^20^	Unspecified	TMD‐associated pain and constrained mouth opening	1.5	12–18	There was a moderate correlation between MRI‐ and US‐measured condylar parameters (*r* = 0.483; *p* < .001 and 0.347; *p* < .001)
Müller et al.^21^	Unspecified	TMD‐associated pain, constrained mouth opening and TMJ palpation	1.5	12	There was a positive relationship between MRI‐ and US‐measured TMJ derangement (*p* = .002)
Tonni et al.^22^	TMD sufferers (*n* = 8) and controls (*n* = 7)	Movement of condylar path, ROM and TMJ palpation	1.5	15	The link between the LPAS measured by the US and MRI was favourable (*r* = 0.623; *p* < .05)
Tonni et al.^23^	TMD sufferers (*n* = 29) and controls (*n* = 28)	Movement of condylar path, ROM and TMJ palpation	1.5	15	The link between the LPAS measured by the US and MRI was found to be positive
Weiss et al.^24^	Unspecified	TMD‐ associated constrained mouth opening	1.5	12.5	There was a negative relationship between MRI‐ and US‐measured acute JIA; however, this link was found to be somewhat moderate in the chronic group
Zwir et al.^25^	Unspecified	Unspecified	1.5	6.7	There was no relationship between MRI‐ and US‐measured synovial parameters.

### 3.1 Statistical results

**FIGURE 3:**
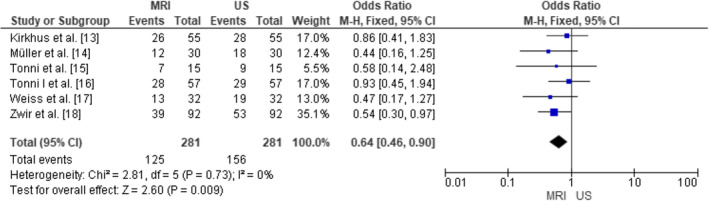
Diagnostic accuracy of magnetic resonance imaging (MRI) as compared to ultrasonography (US) in the selected papers as demonstrated in terms of the calculated OR.

The forest plot in Figure 3 displays the individual study results along with their respective CI and OR. The study by Kirkhus et al.^20^ had 26 events in each group (MRI and US), with an odds ratio of 0.86 and a 95% confidence interval ranging from 0.41 to 1.83. Similarly, the study by Müller et al.^21^ had 12 events in each group, resulting in an odds ratio of 0.44 with a 95% CI from 0.16 to 1.25. Tonni et al.^22^ observed 7 events in each group, yielding an odds ratio of 0.58 and a 95% CI from 0.14 to 2.48. In addition, the study by Tonni et al.^23^ reported 28 events in each group, resulting in an odds ratio of 0.93 with a 95% CI from 0.45 to 1.94. Weiss et al.^24^ had 13 events in each group, with an odds ratio of 0.47 and a 95% CI ranging from 0.17 to 1.27. Lastly, Zwir et al.^25^ reported 39 events in each group, resulting in an odds ratio of 0.54 with a 95% CI from 0.30 to 0.97. A total of 281 events in both MRI and US groups combined were included. The summary odds ratio for all the studies was calculated to be 0.64, with a 95% CI ranging from 0.46 to 0.90. The lower end of the confidence interval indicates that MRI was found to be statistically somewhat better than US in terms of diagnostic accuracy for identifying TMJ DISORDERs. To assess heterogeneity the Chi squared test was also performed, resulting in a value of 2.81 with 5 degrees of freedom (*p* = .73) and the *I* ^2^ test, indicating 0% variability between studies. The low heterogeneity suggests that the studies were relatively consistent in their findings. Moreover, to test the overall effect, the *Z*‐test yielded a value of 2.60 (*p* = .009), indicating that the overall odds ratio was statistically significant.

**FIGURE 4:**
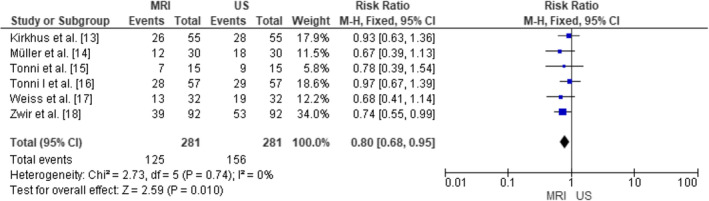
Diagnostic accuracy of magnetic resonance imaging (MRI) as compared to ultrasonography (US) in the selected papers as demonstrated in terms of the calculated relative risks (RRs).

The forest plot in Figure 4 displays the individual study results along with their respective CI and RR. The study by Kirkhus et al.^20^ had 26 events in each group (MRI and US), with a relative risk of 0.93 and a 95% confidence interval ranging from 0.63 to 1.36. Similarly, the study by Müller et al.^21^ had 12 events in each group, resulting in a relative risk of 0.67 with a 95% CI from 0.39 to 1.13. Tonni et al.^22^ observed 7 events in each group, yielding a relative risk of 0.78 and a 95% CI from 0.39 to 1.54. In addition, the study by Tonni et al.^23^ reported 28 events in each group, resulting in a relative risk of 0.97 with a 95% CI from 0.67 to 1.39. Weiss et al.^24^ had 13 events in each group, with a relative risk of 0.68 and a 95% CI ranging from 0.41 to 1.14. Lastly, Zwir et al.^25^ reported 39 events in each group, resulting in a relative risk of 0.74 with a 95% CI from 0.55 to 0.99. Again, a total of 281 events in both MRI and US groups combined were included. The summary relative risk for all the studies was calculated to be 0.80, with a 95% CI ranging from 0.68 to 0.95. The lower end of the confidence interval indicates that MRI was found to be statistically somewhat better than US in terms of diagnostic accuracy for identifying TMJ DISORDERs. To assess heterogeneity, we also performed the Chi squared test, resulting in a value of 2.73 with 5 degrees of freedom (*p* = .74) and the *I* ^2^ test, indicating 0% variability between studies. The low heterogeneity suggests that the studies were relatively consistent in their findings. Also, to test the overall effect, the *Z*‐test yielded a value of 2.59 (*p* = .010), indicating that the overall relative risk was statistically significant.

Variation in diagnostic assessment of US and MRI was noted with differences in ORs (0.44 to 0.93) and RRs (0.67 to 0.97) observed. These differences could be accounted for the varying severity of TMJ disorders, specifications of imaging equipment and diagnostic thresholds utilised to define positive findings. Despite these differences, the pooled effect remained statistically significant presenting results that MRI is superior to ultrasound (US) in the diagnosis of TMJ abnormalities.

## 4 DISCUSSION

The significance of this study is multifaceted, as evident in terms of the comprehensive analysis of diverse findings. The meta‐analysis provided valuable insights into the diagnostic accuracy and efficacy of these imaging modalities for TMJ disorder assessments. By considering the individual study results and summarising them using a fixed‐effects model, the study offers a robust and statistically significant conclusion on the overall effectiveness of MRI as compared to US in diagnosing TMJ disorders. The summary odds ratio of 0.64, with a 95% confidence interval ranging from 0.46 to 0.90, indicates that MRI was associated with a lower risk of TMJ disorder misdiagnosis or false‐negative results compared to US. Likewise, the forest plot for the RR, supported the conclusion that MRI is more effective than US in diagnosing TMJ disorders. The summary relative risk of 0.80, with a 95% confidence interval from 0.68 to 0.95, further corroborates this finding, indicating that MRI was associated with a lower risk of TMJ disorder misdiagnosis compared to US. Additionally, the heterogeneity tests in both forest plots revealed low variability between the studies, indicating that the included investigations were relatively consistent in their findings. The low heterogeneity adds strength to the overall conclusion and suggests that the studies are comparable in terms of their design and population characteristics. Furthermore, the study underscores the need for further research to standardise protocols and enhance the understanding of the diagnostic accuracy and efficacy of US and MRI in TMJ disorder evaluations. This, in turn, can contribute to improved patient care and management of TMJ disorders, ultimately benefiting individuals suffering from these conditions.

In the study by Kirkhus et al.,^20^ the correlation between MRI‐ and US‐measured condylar parameters was moderately positive (*r* = 0.483; *p* < .001). Similarly, Müller et al.^21^ found a positive relationship between MRI‐ and US‐measured TMJ derangement (*p* = .002). In both Tonni et al.^22^ and Tonni et al.,^23^ there was a favourable link between the LPAS measured by US and MRI, with Tonni et al.^22^ reporting a significant correlation (*r* = 0.623; *p* < .05) in TMJ disorder sufferers and controls. On the other hand, Weiss et al.^24^ reported a negative relationship between MRI‐ and US‐measured acute JIA in TMJ disorder‐associated constrained mouth opening. However, this link was found to be somewhat moderate in the chronic group. Lastly, Zwir et al.^25^ observed no significant relationship between MRI‐ and US‐measured synovial parameters.

In the realm of TMJ examination, the detection of intraarticular effusion has garnered attention from researchers. One paper^26^ observed that US continues to hold promise as an alternative imaging modality with commendable accuracy.^4^, ^27^ Notably, the analysis of TMJ capsular width poses a challenging task with a mere 2 mm of precision.^28^ Nevertheless, there exists a consensus among several authors who affirm the efficacy of US in assessing joint effusion and condylar erosion, as indicated by prior studies.^3^, ^29^, ^30^, ^31^, ^32^, ^33^, ^34^ The extant literature highlights the potential utility of US in evaluating the presence of intraarticular effusion within the TMJ area and underscores its reliability in assessing pertinent joint pathologies.

The imperative for a well‐defined and standardised protocol cannot be understated pertaining to TMJ disorder evaluations, as underscored by existing literature.^35^, ^36^, ^37^ However, it is essential to deliberate on the optimal imaging approach, with MRI emerging as a highly precise modality. Yet, judicious consideration should be given to the hierarchy of assessment methods, favouring less‐invasive techniques such as US as the primary option.^11^, ^35^, ^36^, ^37^, ^38^, ^39^ US holds the potential to confirm the diagnosis and bolster sensitivity, as well as enhance the accuracy of positive predictive values.^30^, ^33^, ^34^ The strategic integration of US in the diagnostic pathway can yield valuable insights and complement the utility of MRI. This balanced approach ensures a comprehensive and multifaceted assessment of TMJ conditions, wherein US serves as a vital initial step followed by MRI as a secondary yet crucial confirmatory measure. As such, a strategic amalgamation of these imaging modalities optimises diagnostic accuracy and clinical decision‐making in the evaluation of TMJ disorders.

Despite its valuable findings, this review has several limitations that should be acknowledged. Firstly, the inclusion of a limited number of studies may impact the generalizability of the results. The small sample size and potential publication bias could influence the overall conclusions. Moreover, the heterogeneity of the included studies in terms of sample characteristics, study designs and assessment methods might introduce variability in the results, limiting the ability to draw definitive conclusions. Moreover, the scope of the meta‐analysis did not account for other potentially relevant factors, such as the expertise of the imaging operators or the specific TMJ disorder subtypes being assessed. Neglecting these factors may limit the ability to draw more nuanced conclusions about the efficacy of MRI and US in diagnosing different types of TMJ disorders. Non availability of the records from the database might bring in an element of selection bias, which could affect the generalisability of our findings. But ensuring a robust and thorough search that included various databases, grey literature and conference proceedings helped minimise this risk of bias.

## 5 CONCLUSION

This review provides valuable insights into the diagnostic accuracy and efficacy of US and MRI as imaging modalities. The meta‐analysis indicates that MRI appears to be statistically somewhat better than US in terms of diagnostic accuracy for TMJ disorder identification. The summary odds ratio and relative risk both favour MRI over US, suggesting that MRI is associated with a lower risk of misdiagnosis or false‐negative results in TMJ disorder assessments. However, it is crucial to acknowledge the study's limitations, which may impact the generalizability and robustness of the findings. However, despite these, the review underscores the significance of further research in standardising protocols and conducting larger, well‐designed investigations to establish a clearer understanding of the role of MRI and US in TMJ disorder assessments. By addressing these limitations and considering additional relevant factors, such as specific TMJ disorder subtypes and operator expertise, researchers can enhance the accuracy and applicability of the findings in clinical practice.

## AUTHOR CONTRIBUTIONS

**Giuseppe Minervini:** writing—original draft preparation, writing—review and editing, supervision. **Marco Cicciù:** writing—original draft preparation. **Vincenzo Ronsivalle:** writing—original draft preparation, visualisation. **Mohammed J. Alsaadi:** investigation; data curation. **Mana Alqahtani:** writing—review and editing, supervision. **Marco Cicciù:** writing—review and editing, supervision. **Nasser Raqe Alqhtani:** conceptualization, methodology, software, validation, formal analysis, investigation; data curation. **Mohammad Khursheed Alam:** Conceptualization, methodology, software, validation, formal analysis, investigation; data curation. **Mahmud Uz Zaman:** Conceptualization, methodology, software, validation, formal analysis, investigation; data curation. All authors have read and agreed to the published version of the manuscript.

## FUNDING INFORMATION

This research received no external funding.

## CONFLICT OF INTEREST STATEMENT

The authors declare no conflict of interest.

## Data Availability

All data described in the study are presented in the manuscript. The datasets analysed are available from the corresponding author on reasonable request.
